# Bacterial Endosymbiosis in a Chordate Host: Long-Term Co-Evolution and Conservation of Secondary Metabolism

**DOI:** 10.1371/journal.pone.0080822

**Published:** 2013-12-04

**Authors:** Jason C. Kwan, Eric W. Schmidt

**Affiliations:** Department of Medicinal Chemistry, University of Utah, Salt Lake City, Utah, United States of America; University of Montana - Missoula, United States of America

## Abstract

Intracellular symbiosis is known to be widespread in insects, but there are few described examples in other types of host. These symbionts carry out useful activities such as synthesizing nutrients and conferring resistance against adverse events such as parasitism. Such symbionts persist through host speciation events, being passed down through vertical transmission. Due to various evolutionary forces, symbionts go through a process of genome reduction, eventually resulting in tiny genomes where only those genes essential to immediate survival and those beneficial to the host remain. In the marine environment, invertebrates such as tunicates are known to harbor complex microbiomes implicated in the production of natural products that are toxic and probably serve a defensive function. Here, we show that the intracellular symbiont *Candidatus* Endolissoclinum faulkneri is a long-standing symbiont of the tunicate *Lissoclinum patella*, that has persisted through cryptic speciation of the host. In contrast to the known examples of insect symbionts, which tend to be either relatively recent or ancient relationships, the genome of *Ca.* E. faulkneri has a very low coding density but very few recognizable pseudogenes. The almost complete degradation of intergenic regions and stable gene inventory of extant strains of *Ca.* E. faulkneri show that further degradation and deletion is happening very slowly. This is a novel stage of genome reduction and provides insight into how tiny genomes are formed. The *ptz* pathway, which produces the defensive patellazoles, is shown to date to before the divergence of *Ca.* E. faulkneri strains, reinforcing its importance in this symbiotic relationship. Lastly, as in insects we show that stable symbionts can be lost, as we describe an *L. patella* animal where *Ca.* E. faulkneri is displaced by a likely intracellular pathogen. Our results suggest that intracellular symbionts may be an important source of ecologically significant natural products in animals.

## Introduction

Insects are known to harbor a variety of intracellular symbionts that carry out useful functions, such as synthesizing essential amino acids not found in the host’s diet [1] or conferring resistance to parasitism and disease [2], [3]. In these systems, the bacteria are typically inherited vertically, with the host and symbiont potentially co-evolving for significant time. In recent years, genomic sequencing has revealed that intracellular symbionts in insects undergo a process of progressive genome degradation [1]. Through these studies, a model of symbiont evolution has emerged, whereby the low effective populations of host-restricted symbionts gives rise to a situation where deleterious mutations easily become fixed. The small effective population both weakens purifying selection and reduces the capacity for recombination, leading to progressive gene degradation and genome reduction in a process known as Muller’s ratchet [4]. Concurrently, the intracellular lifestyle of symbionts reduces the need for many functions that would be essential for independent life. Eventually, the loss of DNA repair pathways accelerates the process and increases the rate of genomic drift. Because strongly deleterious mutations are not observed (due to cell loss), the remaining genes in tiny genomes give a clear picture of the symbiont’s role in the relationship, even though their sequences may be suboptimal.

**Figure 1:**
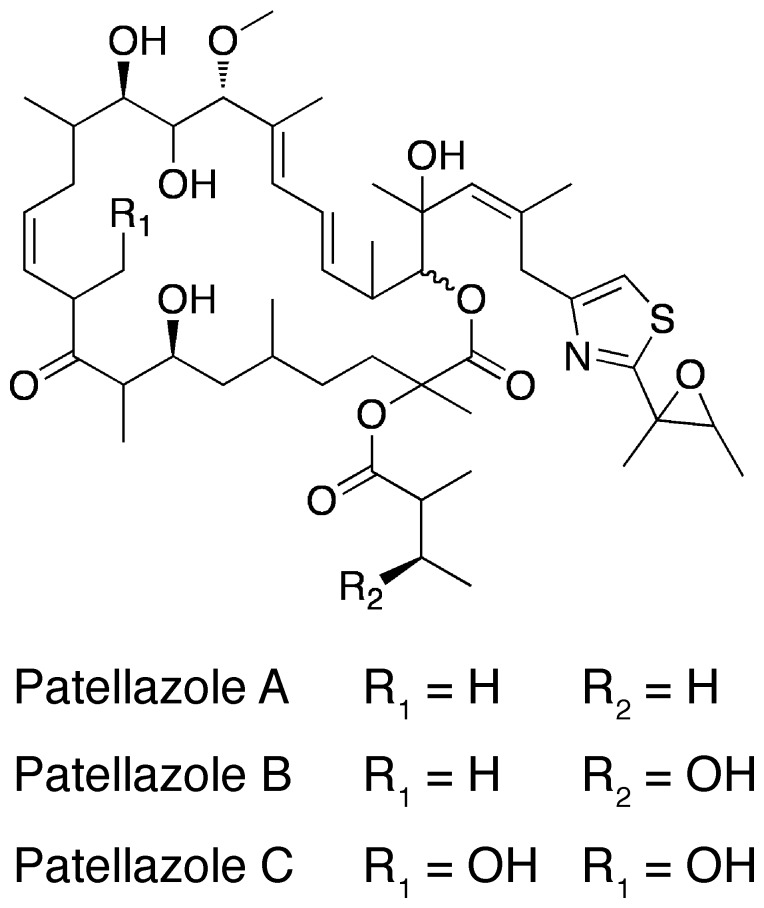
Structures of patellazoles A–C [11], [12], picomolar cytotoxins isolated from the tunicate *Lissoclinum patella* that likely act as chemical defenses.

While intracellular symbionts are common in insects, they have never been found in chordates, such as mammals, where the only intracellular bacteria found are pathogenic [5]. We have been studying a different model of symbiosis, the tunicate *Lissoclinum patella*. Tunicates are sessile marine filter feeders, and as chordates they are the closest extant relatives of the vertebrates [6]. *L. patella* is a colonial tunicate, where groups of individual animals (zooids) live within a common tunic containing shared cloacal cavities. Extruded water from filter feeding, and waste products, are excreted into the shared cloacal cavities [7]. Much of the previous investigations in this system have focused on an extracellular symbiont that resides in the cloacal cavities of *L. patella*, the single-celled cyanobacterium *Prochloron didemni*. Through sequencing, it has been found that this symbiont produces highly modified cyclic peptides termed cyanobactins [8], [9]. More recently it has come to light that *L. patella* has a complex microbiome beyond *P. didemni* [9], and that there are multiple microhabitats within the animal which harbor distinct microbial denizens [10]. In our own efforts, we previously described a novel *α*-proteobacterial symbiont of *L. patella*, and showed that it was associated with the presence of patellazoles [11], [12] (Fig. 1), picomolar toxins that likely serve a protective function for the animal [13]. The genome of this symbiont (accession no. NC_019566), which we termed *Candidatus* Endolissoclinum faulkneri, showed signs of reduction, including low coding density, small gene inventory and loss of the ability for independent replication and division. At the same time the maintenance of the large patellazoles biosynthetic pathway (*ptz*), occupying >10% of the genome’s coding capacity, suggested its central importance to the symbiosis.

Here, we show that *Ca.* E. faulkneri has persisted in its relationship with *L. patella* over an extended period of evolutionary time, illustrating both the age and importance of the *ptz* pathway and the symbiont. Additionally, we show that the nonfunctional ∼40% of the *Ca.* E. faulkneri genome has persisted to the point where it contains few recognizable pseudogenes, showing a temporal separation between degradation and deletion not previously observed. In the one case of an *L. patella* animal that did not possess *Ca.* E. faulkneri, we found that it had been displaced by a likely intracellular pathogen related to known Anaplasmataceae species. This is similar to the phenomenon of symbiont displacement known to occur in insect systems [14]. Collectively, our work captures a novel intermediate stage of genome degradation and reveals similarities to a type of symbiosis previously only well described in insects.

## Results and Discussion

### Potential Cryptic Speciation in the *Ca.* E. faulkneri-Containing Clade of *L. patella*

**Figure 2:**
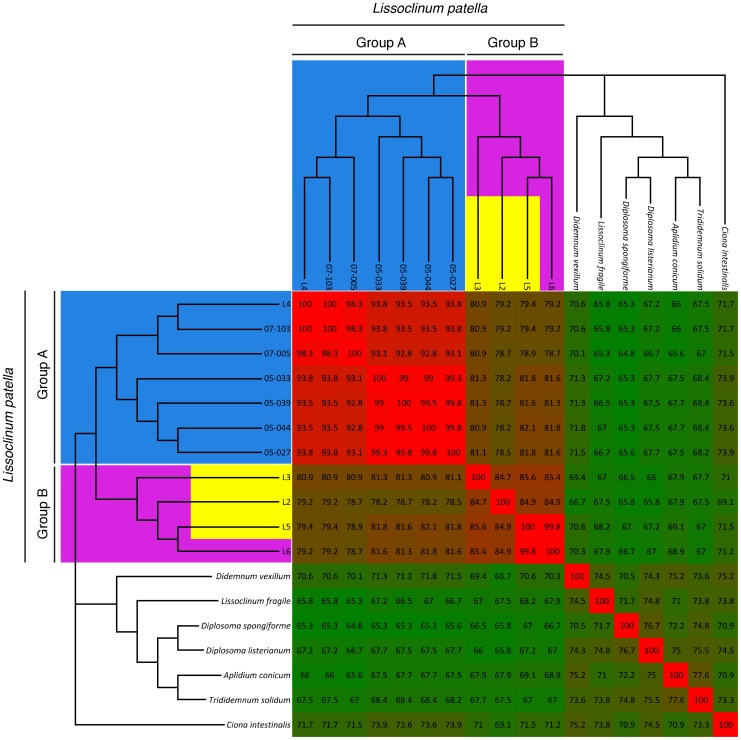
Patellazoles-containing *L. patella* animals form a distinct, divergent clade that may include several cryptic species. Cytochrome *c* oxidase I (COXI) gene nucleotide sequences from various *L. patella* animals were aligned and used to construct a phylogenetic tree. COXI sequences from other didemnid ascidians and *Ciona intestinalis* are included as an outgroup and for comparison, and were obtained from the NCBI database (see Materials and Methods). Animals containing patellazoles are highlighted in yellow, “Group A” is shown in blue and “Group B” is shown in magenta [13].

We previously reported that patellazoles-containing *L. patella* animals formed a distinct clade in phylogenetic trees constructed with 18S rRNA and mitochondrial cytochrome *c* oxidase I (COXI) gene sequences [13]. The intracellular symbiont *Ca.* E. faulkneri, which is the likely source of the patellazoles, is only detected in animals within this clade. We reexamined this clade of *L. patella*, which we previously termed “group B”, and found a high degree of divergence in COXI sequence (Fig. 2). The geographically separated animals L2 (Fiji), L3 (Solomon Islands) and L5 (Papua New Guinea) shared a maximum of 85.6% COXI nucleotide identity (L5–L3). In all animal phyla, infraspecific COXI sequences have rarely been found to diverge greater than 2% [15], and in tunicate lineages cryptic speciation has been claimed based on COXI divergences ranging from 2% to 16.5% [16]–[20]. Although an ascidian molecular clock has not been established, it is known that mitochondrial genomes in this group are highly plastic [21] and divergence rates of 0.5–2.5% per Myr have been estimated [16]–[20]. Using these estimates, divergence of the members of “group B” can be dated to between 6.1 and 31 million years ago, and their degree of divergence suggests cryptic speciation. Although the reproductive compatibility of these animals is unknown, such divergence could be the result of physical separation of these sessile animals along with limited ranges for their gametes and larvae. *L. patella* larvae are known to have a limited range primarily due to predation [22], which also provides an evolutionary rationale for protective compounds such as the patellazoles.

### Conservation of Synteny in *Ca.* E. faulkneri Divergent Strains L2 and L5 Supports Obligate Vertical Inheritance

**Figure 3:**
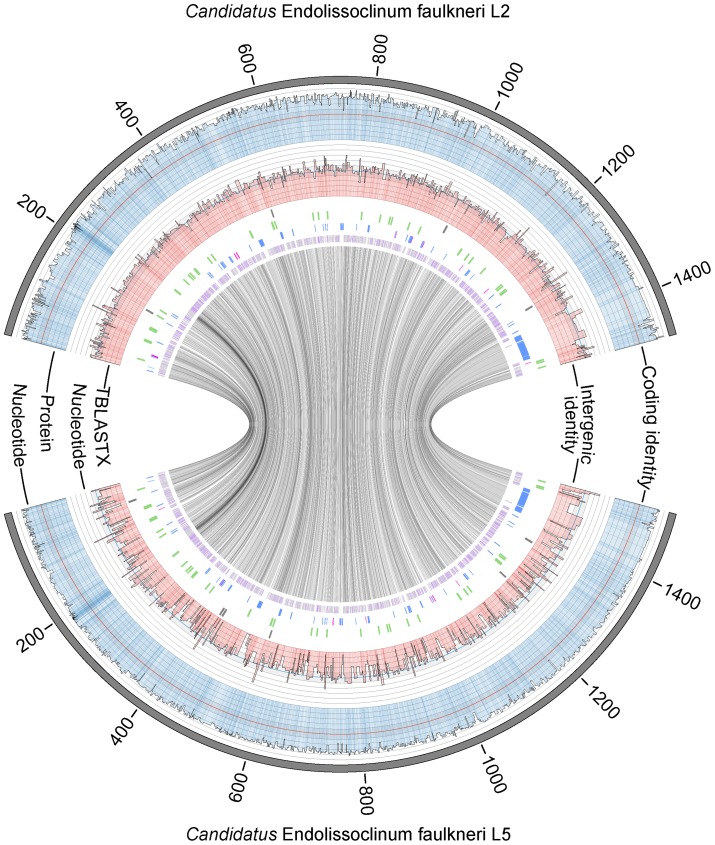
Comparative map of the genomes of *Ca.* E. faulkneri L2 (top) and L5 (bottom). Outer line graphs show protein and nucleotide identities for corresponding coding and noncoding regions (as indicated). In each case the scale runs outwards from 0–100% with lines at intervals of 10%. The 50% line is shown in a contrasting color (red or blue, as appropriate). Note: These graphs are reciprocal and interchangeable, and are plotted against either L2 or L5 for clarity. Immediately inside line graphs are shown the positions of pseudogenes (grey), RNA genes (green), genes that do not have homologs in BAL199, but are shared by both *Ca.* E. faulkneri strains (blue, this category includes all *ptz* genes), and genes that do not have homologs in BAL199, and are unique to one *Ca.* E. faulkneri strain (magenta). The remaining genes (i.e. genes that have a homolog in BAL199), are shown in the innermost track. Note: positioning of the gene categories on different tracks are to allow effective visualization of some positions that overlap due to the pxiel width in the figure. The inner circle shows links between homologous protein coding genes in the two strains of *Ca.* E. faulkneri.

With evidence that the three *L. patella* animals in “group B” were divergent and were possibly cryptic species, we sought to investigate the divergence of the intracellular symbiont *Ca.* E. faulkneri. To this end we carried out shotgun metagenomic sequencing of animal L5, and assembled the chromosome of *Ca.* E. faulkneri *de novo*. The *Ca.* E. faulkneri L5 genome is slightly larger than that of the strain found in L2 (1.51 Mbp vs. 1.48 Mbp, see Fig. 3). These strains share 98.8% nucleotide sequence identity in their 16S rRNA genes, which is above the 97.0% threshold that has been suggested for intraspecies conservation [23]. The closest-related characterized species is *Thalassobaculum litoreum* [80], a member of the family Rhodospirillaceae isolated from coastal seawater in Korea (94% 16S rRNA nucleotide identity to both *Ca.* E. faulkneri L2 and L5). As we noted previously [13], the closest related organism with available genome sequence data is an unclassified and unpublished marine *α*-proteobacterium termed BAL199 (accession no. ABHC00000000, 90% 16S rRNA gene identity to *Ca.* E. faulkneri), whose draft genome is much larger than *Ca.* E. faulkneri (6.1 Mbp) and has much higher GC content (see Table 1).

**Table 1:** Comparison of *Ca.* E. faulkneri, BAL199 and estimated *Ca.* X. pacificiensis genomes.

	L2	L5	BAL199	*Ca.* X. pacificiensis
**Length (Mbp)**	1.48	1.51	6.10	1.04
**% Coding (length)**	57.2	55.6	89.7	77.2
**GC% (coding)**	40.9	41.2	65.7	33.0
**GC% (noncoding)**	24.7	25.9	59.3	28.6
**Protein coding genes**	771	770	6107	956

**Figure 4:**
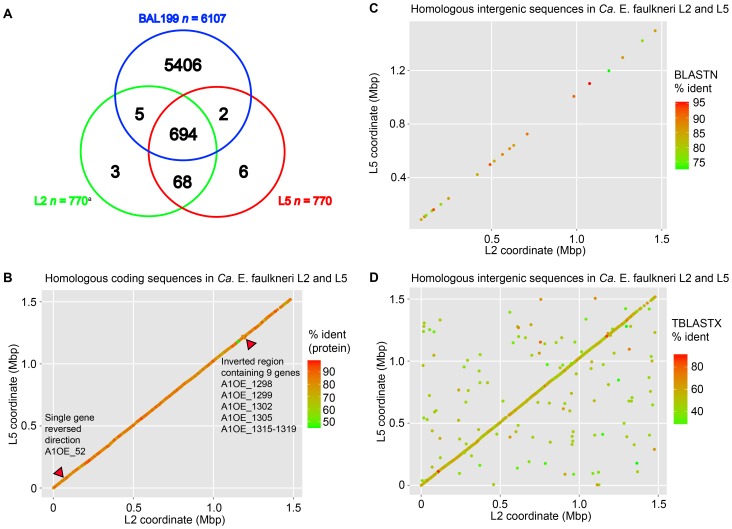
*Ca.* E. faulkneri strains L2 and L5 have very similar gene inventories and exhibit almost complete synteny. Intergenic sequences are highly degraded, but still show a strong syntenic signal, indicating that they were once functional genes. (A) Venn diagram of homologous proteins in *Ca.* E. faulkneri L2, L5 and *α*-proteobacterium BAL199. (B) Synteny plot of proteins in *Ca.* E. faulkneri L2 and L5. (C) Synteny plot of homologous intergenic sequences in *Ca.* E. faulkneri L2 and L5 identified by BLASTN searches. (D) Synteny plot of homologous intergenic sequences in *Ca.* E. faulkneri L2 and L5 identified by TBLASTX searches (see Materials and Methods). aFor the purposes of the Venn diagram, the number of protein coding genes in *Ca.* E. faulkneri is given as 770, because A1OE_1073 and A1OE_1074 are both homologous to P856_600 (see Main Text).

**Figure 5:**
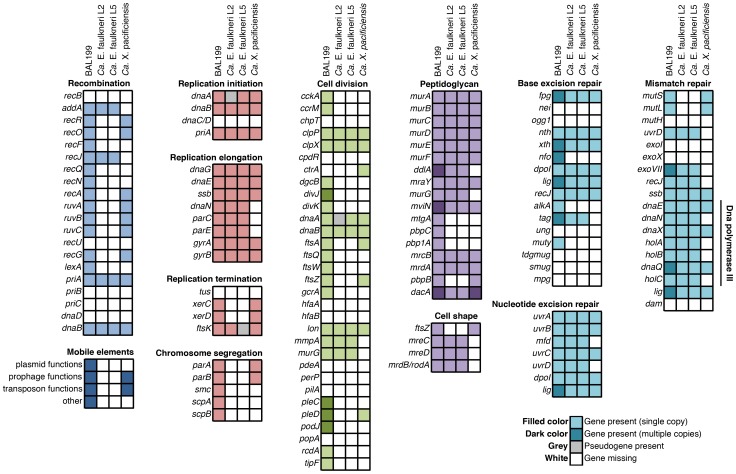
*Ca.* E. faulkneri is missing key genes involved in the processes of recombination, replication, cell division and mismatch repair. Note: some genes are repeated in multiple categories. The BAL199 and *Ca.* X. pacificiensis genomes are not closed, and so genes presented as missing above may possibly be present but not assembled.

**Figure 6:**
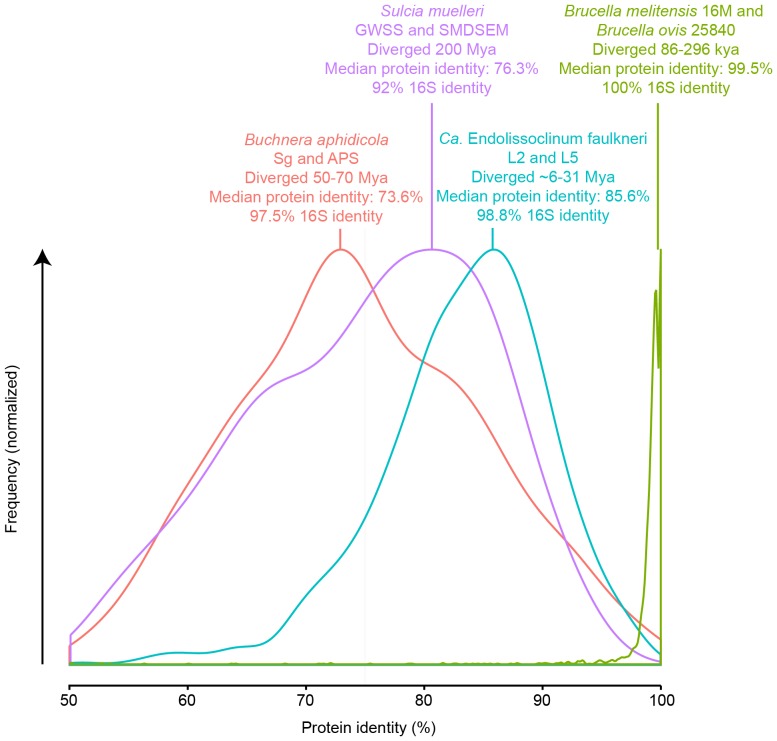
Sequence drift in the genomes of *Ca.* E. faulkneri is on the order of that seen in pairs of endosymbionts known to have diverged tens of millions of years ago. By comparison, a pair of intracellular pathogens that are thought to have diverged before the domestication of their respective hosts exhibits far less sequence drift.

Analysis of the homologs in *Ca.* E. faulkneri L2, L5 and BAL199 reveals that 90.1% of genes found in *Ca.* E. faulkneri have homologs in BAL199, and the remainder are almost all conserved between L2 and L5 (see Fig. 4A). Homologs in the two strains of *Ca.* E. faulkneri diverge significantly (median protein identity 85.6%), yet we found that synteny is almost entirely conserved (see Fig. 4B), with only two small in-place inversions. This is consistent with intracellular symbionts in insects, such as *Buchnera aphidicola* [25] and *Sulcia muelleri* [26], in which genetic isolation and loss of recombination pathways allows complete synteny conservation over hundreds of millions of years, despite significant sequence drift. These symbionts have genomes in a more advanced stage of reduction than *Ca.* E. faulkneri: the ∼641 kbp genomes of *B. aphidicola* contain ∼550 protein coding genes, and the ∼260 kbp genomes of *S. muelleri* contain ∼230 protein coding genes. *B. aphidicola* strains APS and Sg are found in the aphid species *Acyrthosiphon pisum* and *Schizaphis graminum*, respectively [25]; because these symbionts are strictly vertically transmitted, their divergence follows that of the hosts, estimated to have occurred 50–70 million years ago [25]. Similarly, the *S. muelleri* strains GWSS (host: glassy-winged sharpshooter) and SMDSEM (host: cicada) are estimated to have diverged at least 200 million years ago [26]. As with *B. aphidicola* and *S. muelleri*, *Ca.* E. faulkneri has lost *recA* (see Fig. 5), and we found no evidence of mobile elements in the genomes of L2 or L5. We compared the level of divergence in the protein coding genes of L2/L5 with that of other strain pairs with dated divergence (Fig. 6). *Ca.* E. faulkneri strains exhibited an intermediate level of divergence between, on one extreme, *S. muelleri* [26] and *B. aphidicola* [25], and a more recent speciation in strains of *Brucella* [27]. *Brucella melitensis* 16M and *Brucella ovis* 25840 are intracellular pathogens of livestock, whose divergence has been dated to 86,000–296,000 years ago, which presumably occurred in wild animal populations prior to domestication [27]. Taken together, the divergence and synteny of L2 and L5 suggest that the two strains have been genetically isolated since the divergence of their hosts, roughly concurrently with the loss of recombination pathways, as evidenced by the two observed inversion events, which presumably occurred shortly before the capability was lost.

### *Ca.* E. faulkneri Strains L2 and L5 Contain Only a Small Number of Recent Pseudogenes

**Figure 7:**
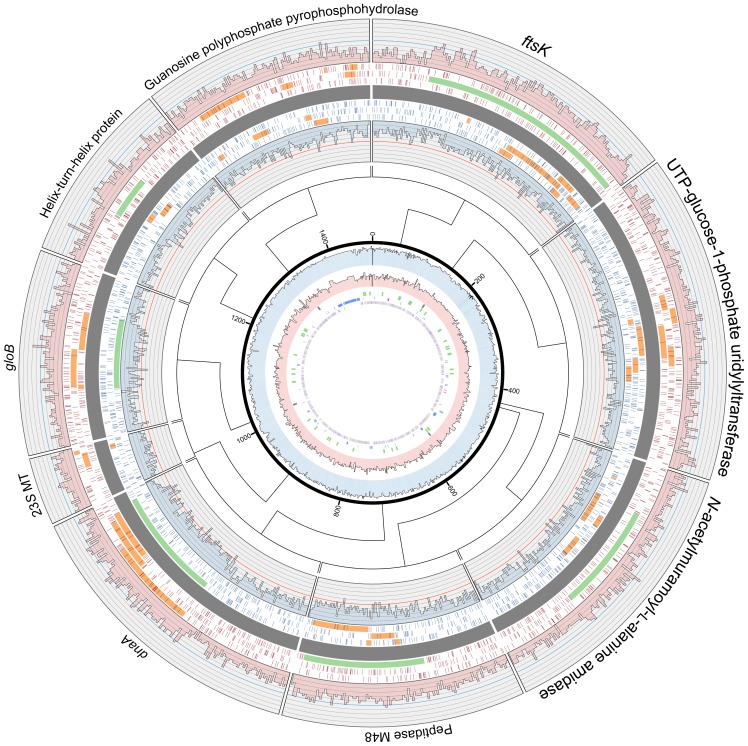
Recent pseudogenes in *Ca.* E. faulkneri L2 and L5 contain many frame-shifts and in-frame stop codons, and exhibit GC content higher than most intergenic sequences (see Table 1). Syntenic regions in *Ca.* E. faulkneri L2 (red, outside) and L5 (blue, inside) are shown aligned, with heatmaps showing the locations of stop codons in three frames (for simplicity, each region is shown in the same orientation, where the forward coding direction is clockwise), and GC content shown in histograms. Intact genes are shown in light green, while pseudogenous regions that show homology in BLASTX/TBLASTN searches are shown in light orange. The locations of these regions are shown in relation to the L2 chromosome (center).

**Figure 8:**
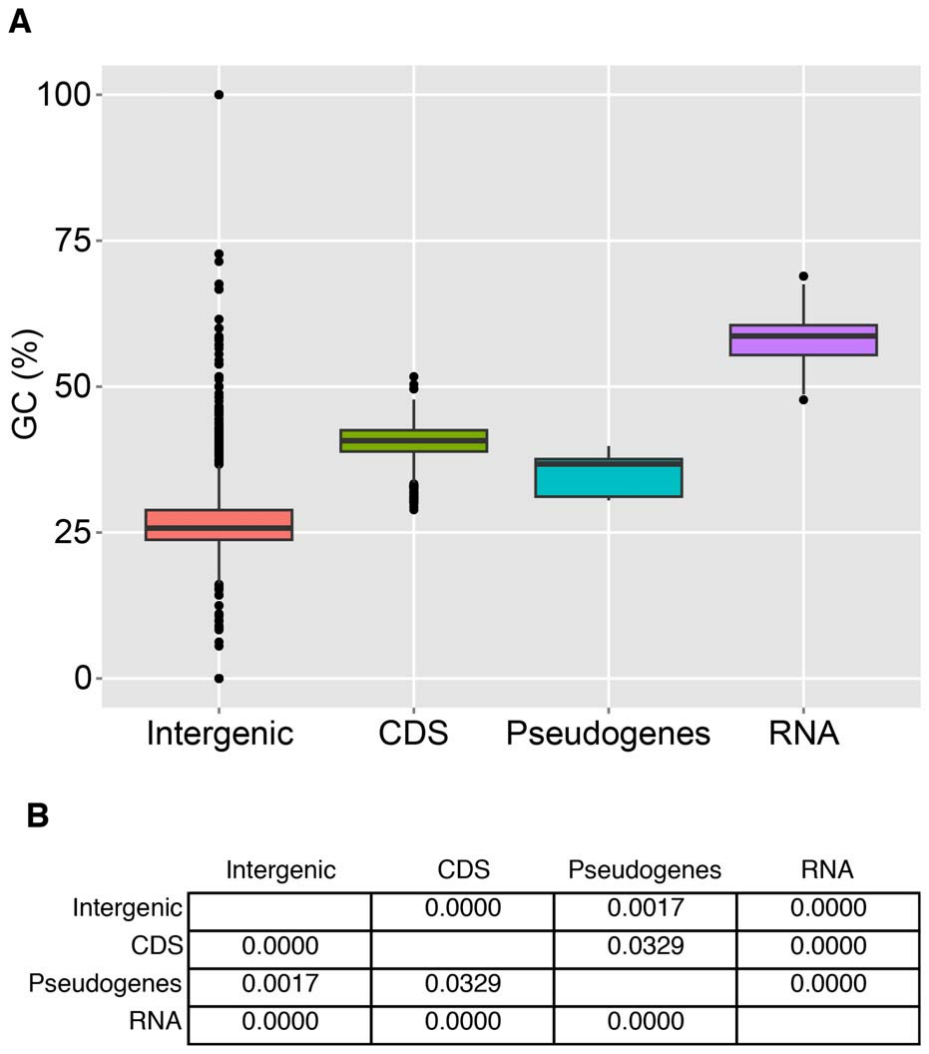
The *Ca.* E. faulkneri genome is made up of regions with different GC content. The GC content of discrete regions (for example, all coding sequences) from the genomes of both *Ca.* E. faulkneri strains were tabulated and used to create the boxplot shown in (A). The groups were determined to be different from one another by ANOVA followed by Tukey HSD (see Materials and Methods). The individual *p* values for the pairwise comparisons are shown in (B).

The genomes of *Ca.* E. faulkneri L2 and L5 both have a very low coding density (57% versus a bacterial average of 90% [28]). This is similar to the early stages of genome reduction following host-restriction, which are characterized by a proliferation of pseudogenes (and therefore low coding density), and pervasive genome rearrangements, with only modest genome reduction [1]. In previously described low-density genomes, large numbers of pseudogenes have been found. For instance, 972 pseudogenes were described in the 4.2 Mbp *Sodalis glossinidius* genome (coding density 51%) [29], and 550 pseudogenes were found in the 2.8 Mbp *Serratia symbiotica* genome (coding density 60.9%) [30]. In these cases, symbiosis was established fairly recently. For example, a rice weevil symbiont related to *S. glossinidius* called SOPE has a 4.5 Mbp genome with 1194 pseudogenes [31]. Recently a closely related free-living pathogen (HC) was discovered and appears to have diverged from SOPE approvimately 28,000 years ago [31]. We searched for pseudogenes in both strains of *Ca.* E. faulkneri by using intergenic sequences as queries in BLASTX searches against the NR database, and also by using orphan genes in both strains as queries in searches against their partner. These efforts yielded strikingly few identifiable pseudogenes (see Table 2). All of these pseudogenes are characterized by numerous frame shifts and in-frame stop codons (see Fig. 7). Their putative functions are in line with the types of losses observed in other intracellular symbionts: replication, gene regulation, stress response and peptidoglycan degradation. The remaining intergenic sequences display an AT content much lower than that of coding sequences (see Table 1 and Figure 8), in contrast to the intergenic/pseudogene regions of the *S. glossinidius* genome, which in our analysis was very similar to coding regions (accession NC_007712, 56.2% for coding vs. 53.1% for noncoding). The higher AT content of intergenic sequences likely reflects AT mutation biases acting disproportionately on nonfunctional sections of the genome, as part of a general trend of increased AT content in progressively reduced genomes [1]. The intergenic sequences of *Ca.* E. faulkneri are extremely degraded – in BLASTN searches, we were only able to find 21 hits among hundreds of intergenic sequences (see Fig. 4C), although notably these hits were all syntenic. Nevertheless, in reciprocal TBLASTX searches they still show a strong syntenic signal (see Fig. 4D), even with preservation of the two inversions seen in the synteny plot of coding regions (Fig. 4B). This strongly suggests that these intergenic sequences were once genes that underwent an extended period of time where synonymous mutations were favored. This finding is unique amongst symbiont genomes and could suggest that the process of pseudogene formation proceeds in distinct stages, with sequence degradation preceding deletion. Other examples of long-term obligate symbionts in insects all have much smaller genomes where the majority of pseudogenes have already been deleted, and their symbiotic lifestyles date back to much earlier times (genome sizes 140–700 kbp, 40–270 million years ago [14]). Further work would be required to determine if the highly-degraded sparse genome reduction stage is restricted to certain types of organism or systems, or whether it represents an intermediate stage held through which the more reduced insect symbionts have passed.

**Table 2:** Pseudogenes in *Ca.* E. faulkneri L2 and L5.

L2 Coordinates	L5 Coordinates	Strand	Nearest homolog	Function
	52194–53458	+	A1OE_48 ftsK/SpoIIIE family protein	Replication
153326–154049	155506–156085	+	UTP-glucose-1-phosphate uridylyltransferase	Glucose
			[alphaproteobacterium BAL199] (WP_007680598)	metabolism
	431976–432886	−	A1OE_465 *N*-acetylmuramoyl- L-alanine amidase	Peptidoglycan
			family protein	degradation
	439025–440102	+	A1OE_470 peptidase M48 family protein	Heat shock
				protein
722626–724001		+	P856_442 chromosomal replication initiator	Replication
			protein DnaA	
903089–903300		+	23S rRNA (uracil-5-)-methyltransferase, partial	Ribosomal
			[*Methylobacterium extorquens*] (WP_003607533)	structure
1019673–1020560		+	P856_614 hydroxyacylglutathione hydrolase GloB	*β*-lactamase
	1237554–1237896	−	A1OE_1333 helix-turn-helix family protein	Regulation
1317241–1318045	1349258–1350574	+	Guanosine polyphosphate pyrophosphohydrolase	Stringent
			/sythetase alpha proteobacterium BAL199	response
			(WP_007669831)	

**Figure 9:**
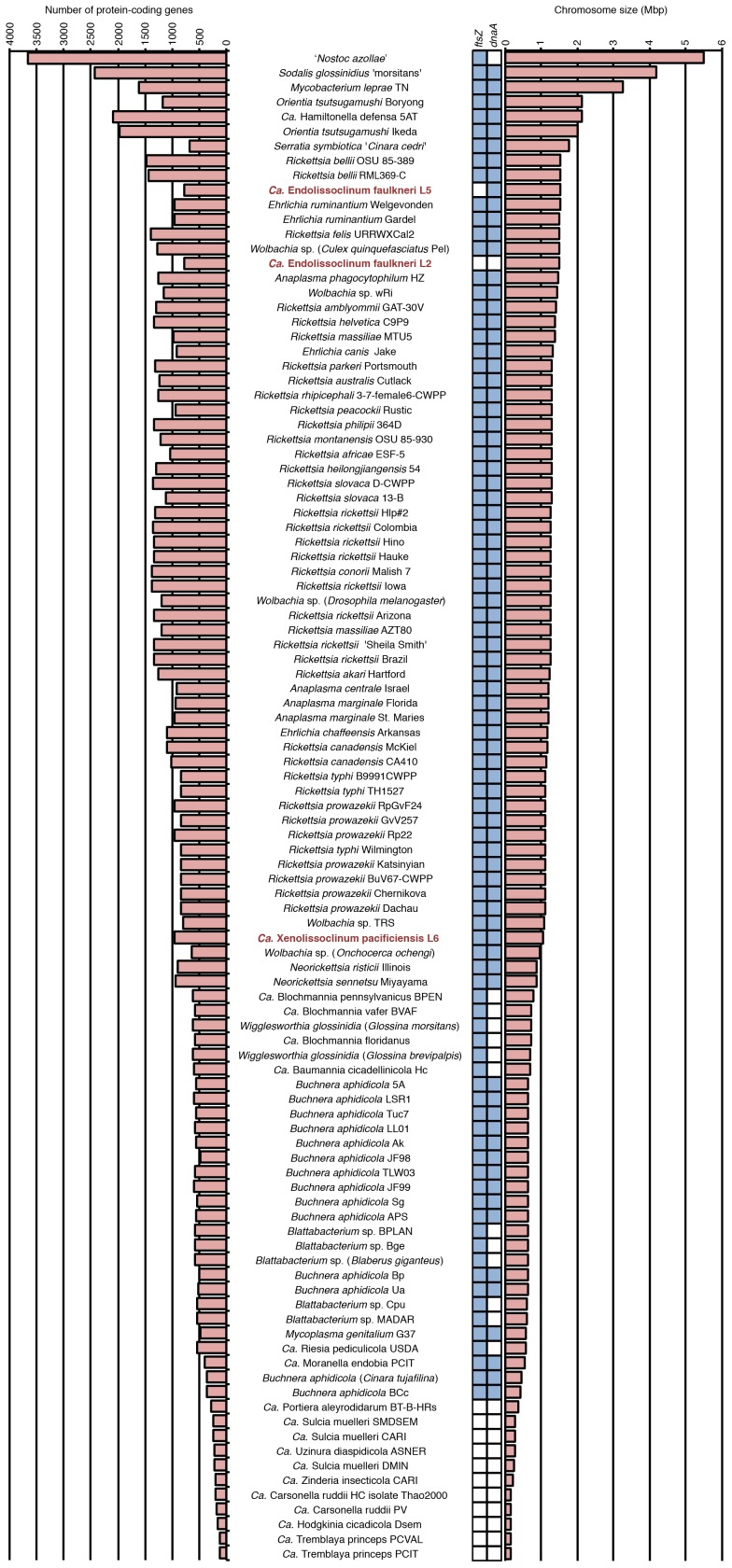
*Ca.* E. faulkneri strains have low coding density and possess the largest genomes lacking *ftsZ*, and *Ca.* E. faulkneri L2 has the largest genome also lacking *dnaA*. A set of symbionts and pathogens that live intracellularly and/or show signs of genome reduction are shown, in descending order of genome size. Shaded blue squares indicate the presence of *ftsZ* or *dnaA*. Note: All genomes included above are completed except for *Ca.* X. pacificiensis where the genome size plotted reflects the sum of contig sizes in the draft genome.

Analysis of the gene inventories of L2 and L5 showed loss of key genes involved in chromosomal replication, DNA mismatch repair and cell division (Fig. 5). For instance, the replication initiator *dnaA* [32] is missing in L2, whereas *ftsK*, which is involved in the termination of replication, is missing in L5. These genes are detectable as pseudogenes (see Table 2) and intact ORFs in the corresponding strain (see Table 3), suggesting that their loss was recent. This is supported by the GC content of the pseudogenes, which are at an intermediate GC content, significantly different from both coding and intergenic regions (see Table 1, Fig. 7 and Fig. 8). Other central genes are missing in both L2 and L5, for instance the almost universally conserved protein FtsZ [33], which has a central role in bacterial cell division, forming the Z-ring that divides an elongated rod cell in the center. To the best of our knowledge, *Ca.* E. faulkneri L2 possesses the largest genome lacking both *dnaA* and *ftsZ*, and *Ca.* E. faulkneri L5 possesses the largest genome lacking *ftsZ* (see Fig. 9). It should be noted that in our analysis we did not include members of the Chlamydieae and Planctomycetes lineages, which do not possess *ftsZ* but may use other methods to produce a peptidoglycan septum [33]. The only other example of loss of these genes in early-stage symbiosis is the cyanobacterium ‘*Nostoc azollae*’, an extracellular symbiont of the plant *Azola filiculoides* [34]. This cyanobacterium still has a large genome with low-coding density and many pseudogenes, including *dnaA*, but it still possesses *ftsZ* [35]. An *ftsZ* pseudogene could not be found in either strain of *Ca.* E. faulkneri despite extensive searches, suggesting that it was not lost recently. Both strains are missing the key genes *mutSLH*, involved in the initial recognition of DNA mismatches prior to repair [35] (see Fig. 5). The nucleotide excision repair pathway appears to be complete, and the base excision repair pathway contains a similar gene complement to BAL199, suggesting that while DNA damage caused by UV radiation may be repairable in *Ca.* E. faulkneri, mismatches from replication errors and mutations will become fixed [35]. The loss of DNA repair pathways is thought to be one of the driving forces for increased rate of evolution observed in some intracellular symbionts [1].

**Table 3:** Orphan genesa in *Ca.* E. faulkneri strains L2 and L5.

Strain	Locus tag	Annotation	Homolog in BAL199	corresponding strain?
L2	A1OE_1333	helix-turn-helix	WP_007672880	Yes
		family protein		
L2	A1OE_1515	lipid A biosynthesis N-	WP_007668018	No
		terminal domain protein		
L2	A1OE_465	*N*-acetylmuramoyl-L-	WP_007674464	Yes
		alanine amidase family protein		
L2	A1OE_470	peptidase M48 family protein	WP_007674472	Yes
L2	A1OE_48	*ftsK*/SpoIIIE	WP_007677281	Yes
		family protein		
L2	A1OE_1223	hypothetical protein		No
L2	A1OE_977	hypothetical protein		No
L2	A1OE_1030	hypothetical protein		No
L5	P856_442	chromosomal replication	WP_007679288	Yes
		initiator protein DnaA		
L5	P856_69	hypothetical protein		No
L5	P856_611	hypothetical protein		No
L5	P856_614	hydroxyacylglutathione		Yes
		hydrolase GloB		
L5	P856_659	hypothetical protein		No
L5	P856_249	hypothetical protein		No
L5	P856_500	hypothetical protein		No
L5	P856_13	hypothetical protein	WP_007677288	No

a These are annotated genes in one *Ca.* E. faulkneri strain that are not found in the corresponding strain.

**Figure 10:**
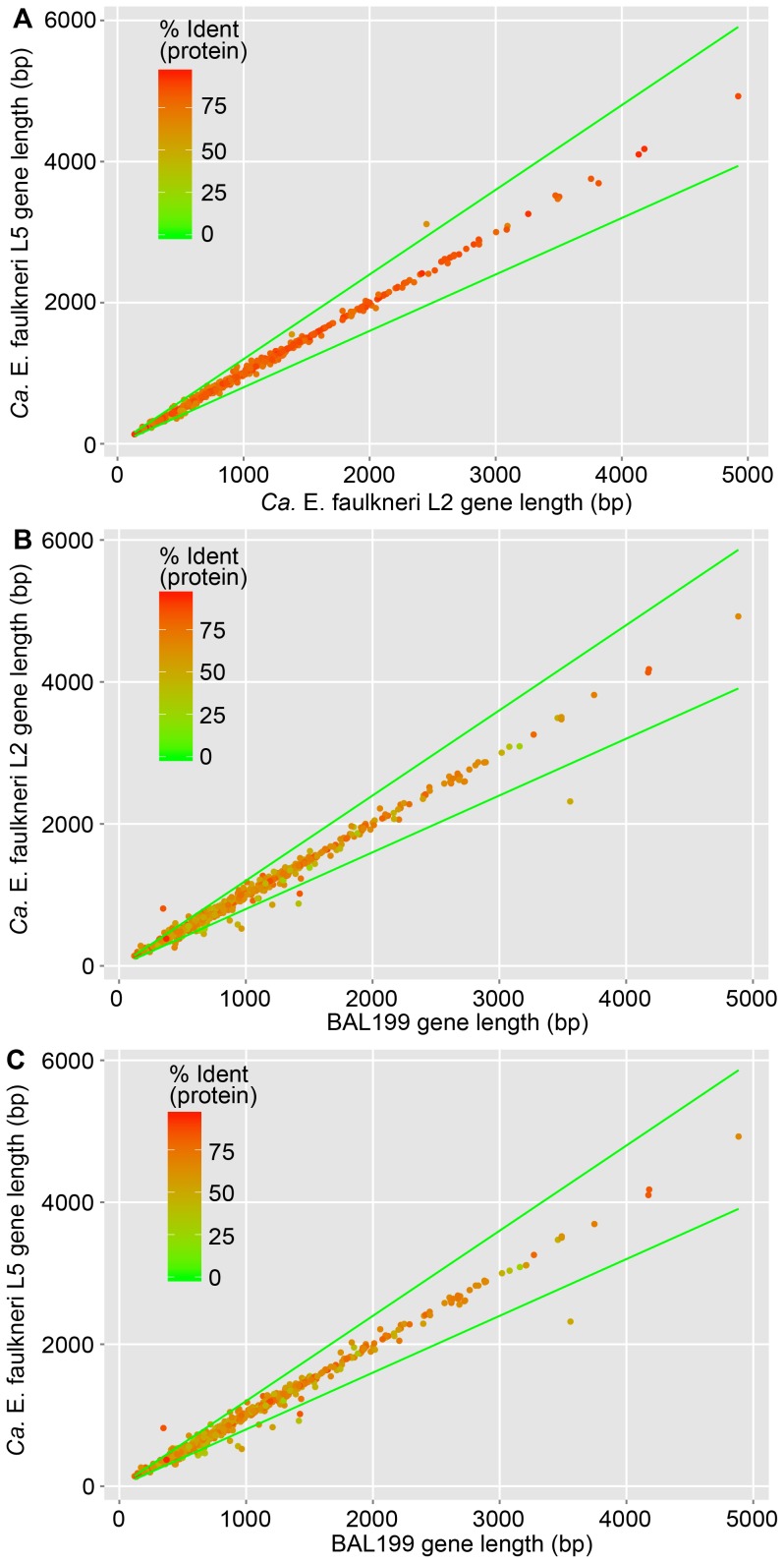
Only one gene varies in length by more than 20% in *Ca.* E. faulkneri strains L2 and L5, and only a few are truncated by more than 20% versus their homologs in BAL199. Comparisons shown: (A) *Ca.* E. faulkneri L2 and L5, (B) *Ca.* E. faulkneri L2 and BAL199, and (C) *Ca.* E. faulkneri L5 and BAL199. Green lines denote a tolerance of ±20% of the *x*-axis variable. Note: the large PKS genes in the *ptz* pathway are omitted in order to avoid skewing the scale of axes in (A).

The vast majority of extant genes are shared between *Ca.* E. faulkneri L2 and L5 (see Fig. 4A). Of the small number of orphan genes in each strain, the majority are found as pseudogenes in the corresponding strain or else are short hypothetical genes (see Table 3). We found only one instance where homologs differed in length by more than 20%, a criterion that has been used to identify pseudogenes [36] (see Fig. 10). The one instance of truncation was a multiefflux transporter in L5 (P856_600), which was rendered into two proteins in L2 (A1OE_1073 and A1OE_1074) by a frameshift. When comparing lengths of *Ca.* E. faulkneri proteins to their homologs in BAL199, very few differed by more than 20% (see Fig. 10 and Table 4). These shortened ORFs may in fact be pseudogenes that have undergone deletions and have not yet been disrupted by in-frame stop codons. Collectively, the low number of identifiable pseudogenes and truncated ORFs would suggest that the majority of annotated protein coding genes are not pseudogenes. However, at least some of these genes may still be nonfunctional, even if transcribed. For instance, although both strains of *Ca.* E. faulkneri possess the vast majority of peptidoglycan biosynthesis and rod-shape determining genes (except for *ftsZ*, see Fig. 5), cells are irregular globules [13] that may lack a cell wall. Such irregular cell shape is only seen elsewhere in symbionts with genomes <250 kbp that are missing almost all genes involved in cell envelope biosynthesis and that “appear to lack a cell wall” [1]. Likewise, although intracellular bacteria typically lose lipopolysaccharide pathways [37], *Ca.* E. faulkneri maintains the genes required for UDP-*N*-acetyl-D-glucosamine synthesis (*glmS*, *mrsA* and *glmU*) and the majority of genes required for synthesis of the LPS precursor Kdo_2_-lipid A (*lpxACDHBKLM*, *kdsACB*, and *kdtA*) [38]. However, genes required for the later steps in LPS synthesis are missing.

**Table 4:** *Ca.* E. faulkneri genes that are more than 20% shorter than their homolog in BAL199 in at least one strain.

L2 Gene	% Difference in length	L5 Gene	% Difference in length	Annotation
A1OE_924	−45.7%	P856_522	−45.7%	rare lipoprotein A
A1OE_1441	−41.1%	P856_761	−29.5%	*rpoZ* DNA-directed
				RNA polymerase, omega
				subunit
A1OE_538	−38.1%	P856_302	−35.0%	*ccmI*
				cytochrome c-type
				biogenesis protein
A1OE_1235	−37.8%	P856_668	−39.7%	conserved
				hypothetical protein
A1OE_177	−34.9%	P856_104	−34.8%	putative glycosyl
				transferase
A1OE_432	−32.7%	P856_255	−20.2%	*ccmA* cell envelope
				outer membane
				protein
A1OE_399	−29.7%	P856_237	−30.4%	*rspP* ribosomal
				protein S16
A1OE_1503	−28.8%	P856_799	−31.3%	*rluB*
				pseudouridine
				synthase
A1OE_720	−28.6%	P856_405	−28.6%	putative pyruvate
				dehydrogenase E1
				component, beta
				subunit
A1OE_72	−26.8%	P856_52	−26.8%	hemolysin C
A1OE_1058	−23.1%	P856_590	−31.1%	colicin V production
				family protein
A1OE_986	−22.1%	P856_554	−26.8%	*tatB*
A1OE_347	−19.7%	P856_211	−20.7%	*rplI* ribosomal
				protein L9
A1OE_1508	−13.6%	P856_803	−29.5%	hypothetical protein
A1OE_741	−13.6%	P856_420	−21.3%	*hisC* histidinol-phosphate
				aminotransferase

Recent work in insects suggests that when evolutionary pressures change (for example after the acquisition of a second symbiont by the host), a new round of genome degradation and pseudogenization can be precipitated, even in tiny genomes [26], [39]. Our analysis suggests that *Ca.* E. faulkneri has been under unchanging pressures, at least since the divergence of strains L2 and L5, because we see very few recent pseudogenes and the majority of extant genes are conserved in both L2 and L5. In our estimation the two sequenced strains of *Ca.* E. faulkneri diverged somewhere between 6 and 31 million years ago, and the conservation of the gene inventories between the strains suggests that most of the degraded genes were already in a state of decay at the point of divergence. This is further supported by the lingering similarity of intergenic regions by TBLASTX but *not* BLASTN. Very few cases of gene decay since that time are readily apparent, and include *ftsK* and *dnaA*, both important genes in chromosomal replication. It is remarkable that *ftsZ*, which is missing in both strains, was not identified as a pseudogene, given its maintenance in much smaller genomes [1] and its central role in both cell division and maintenance of rod shape.

### The *ptz* Cluster was Present before the Divergence of L2 and L5

Genes within the *ptz* pathway are not found in one distinct locus, but are in fact scattered throughout the genome (see Fig. 3). We previously suggested [13] that this scattering was a result of an ancestral *ptz* gene cluster being present in the genome of *Ca.* E. faulkneri before a period of pervasive genome rearrangements seen in early stages of host-restriction [1]. When comparing the entire *ptz* pathway in L2 and L5, we observe a similar degree of divergence as with other protein-coding genes in these strains (∼85%, see Table 5) and these genes are part of the almost complete conservation of synteny maintained since the divergence of L2 and L5. This strongly suggests that the acquisition of the *ptz* pathway dates to before the divergence of *Ca.* E. faulkneri L2 and L5.

**Table 5:** Comparison of *ptz* genes in *Ca.* E. faulkneri L2 and L5.

Gene	Functiona	Alignment	Protein	Nucleotide
		length (bp)	identity (%)	identity (%)
*ptzA*	PKS	9,357	86.2	86.9
*ptzB*	PKS	3,753	85.4	87.0
*ptzC*	PKS	15,105	84.7	86.3
*ptzD*	PKS	19,659	84.6	85.9
*ptzE*	PKS	14,400	81.7	84.9
*ptzF*	PKS	10,710	83.4	85.6
*ptzG*	ECH	747	93.6	90.1
*ptzH*	KS	1,224	82.8	84.2
*ptzI*	HMGS	1,311	93.0	90.8
*ptzJ*	ECH	795	90.2	87.2
*ptzK*	AT2	984	86.3	86.2
*ptzL*	AT1	873	83.8	86.3
*ptzM*	P450	1,347	92.2	88.8
*ptzN*	P450	1,308	90.0	88.8
*ptzO*	DCR	804	91.8	87.2
*ptzP*	Ox	1,344	79.4	83.9
*ptzQ*	ER	1,524	86.2	86.8
*ptzR*	C	1,326	85.7	87.6

a Abbreviations: AT1, *trans*-acting acyltransferase; AT2, proofreading acyltransferase [22]; C, condensation; DCR, 2,4-dienoyl-CoA-reductase; ECH, enoyl-CoA-reductase; ER, enoyl-reductase; HMGS, 3-hydroxy-3-methylglutaryl-CoA-synthase; KS, ketosynthase; Ox, thiazoline oxidase; P450, cytochrome P450; PKS, polyketide synthase.

**Figure 11:**
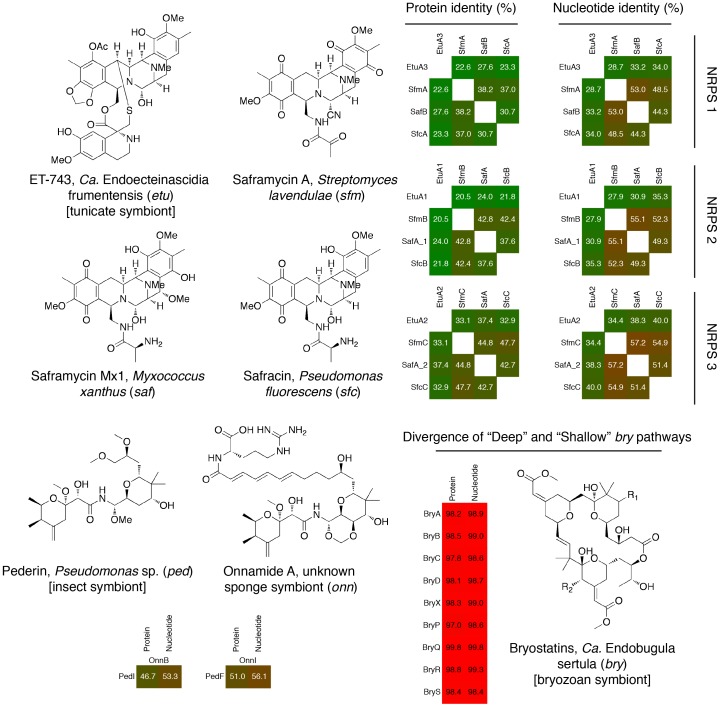
Divergence in biosynthetic pathways which produce natural products with related structures.

Although the *ptz* pathway has diverged significantly since the common ancestor of *Ca.* E. faulkneri L2 and L5, the structure of the patellazoles remains the same. In samples L2, L3 and L5, we previously detected patellazoles A and B at the same retention times and with masses within 5 ppm of calculated values for their published molecular formulas [13]. There are examples of natural product pathways that produce very similar structures despite significant sequence divergence. This situation usually arises where an ecologically important pathway is frequently transferred horizontally between bacteria. In such cases related pathways may be isolated from each other following a horizontal transfer if the descendants of the donor and recipient strain adapt to different environments. For example, the anti-cancer drug ET-743/trabectedin (brand name Yondelis) is a minor groove DNA-alkylating agent [40] produced by *Ca.* Endoecteinascidia frumentis, a symbiont of the tunicate *Ecteinascidia turbinata* [41]. The nonribosomal peptide synthetase (NRPS) proteins of the ET-743 pathway are related to others found in free-living bacteria such as *Streptomyces lavendulae* (actinobacteria), *Myxococcus xanthus* (*δ*-proteobacteria) and *Pseudomonas fluorescens* (*γ*-proteobacteria). Collectively, these pathways make diversified structures with related core motifs (see Fig. 11). The NRPS proteins that make up these pathways are highly divergent, with protein and nucleotide identities in the region of 20–50% (see Fig. 11). Similarly pederin and the onnamides (see Fig. 11) have related structures but are produced in very different environments. Pederin is found in *Paederus* spp. beetles, and acts as a chemical defense made by a *Pseudomonas* sp. symbiont [42], [43], while onnamides are found in several marine sponges [44], [45]. The pederin pathway polyketide synthase (PKS) proteins PedI and PedF produce products analogous to the onnamide pathway proteins OnnB and OnnI, respectively. These protein pairs maintain an identity of 50% (see Fig. 11).

The *ptz* pathway genes in *Ca.* E. faulkneri strains L2 and L5 are not as divergent as the cases of ET-743 and pederin pathway homologs. However, the divergence of *ptz* is notable as the two variants make the same compounds, rather than analogs, and they have been maintained in two descendants of a common ancestor. The closest analog of this system is the *γ*-proteobacterium *Ca.* Endobugula sertula, which produces the cytotoxic bryostatins within its host, the bryozoan *Bugula neritina*. *B. neritina* has three subspecies, known as “Deep”, “Shallow” and “Northern”, that occupy different habitats [46]. Bryostatins are only detected in the “Deep” and “Shallow” subspecies, with each producing the same core macrocyclic polyketide structure, but with different side chains [46]. Although the full genome of *Ca.* E. sertula has not been sequenced, there is evidence that genome rearrangements have occurred since the divergence of the “Deep” and “Shallow” forms, because while the known genes in the *bry* pathway are in a single locus in “Shallow”, they are found on separate clones in “Deep” [46]. Nevertheless, gene sequences in the two pathways are still highly similar (see Fig. 11), in contrast to the *ptz* pathway.

Our work supports the idea that natural products can be the basis of stable and long-lived symbiotic relationships. Although this idea has developed over several years based on the examples outlined above, generally little is known about the evolutionary history of the producing symbionts. In the case of the pederin pathway there is evidence that natural product pathways have been horizontally acquired relatively recently based on sequence analysis [47]. In this case the symbiosis may also be relatively recent, although an age has not been estimated. Recently a symbiont of the Asian citrus psyllid *Diaphorina citri*, *Ca.* Profftella armatura, was found to contain a functional polyketide pathway (termed *dip*) related to that of pederin [48]. *Ca.* P. armatura has a 460 kbp genome (89.5% coding density), and thus it is in a more advanced stage of genome reduction than *Ca.* E. faulkneri. There is some evidence that this symbiont serves a nutritional function in addition to synthesizing the polyketide diaphorin, and thus it is unclear how old *dip* is or when it was acquired by the symbiont, since nucleotide analysis does not suggest a recent acquisition. Because symbionts with reduced genomes lose the ability to accept horizontally transferred DNA, *dip* likely dates to early on in the symbiotic relationship. Together with *Ca.* E. faulkneri, the discovery of *Ca.* P. armatura suggests that large natural product pathways can be conserved in obligate symbionts over vast evolutionary time scales.

### A New Anaplasmataceae Species is Found in *L. patella* L6 in Place of *Ca.* E. faulkneri

**Figure 12:**
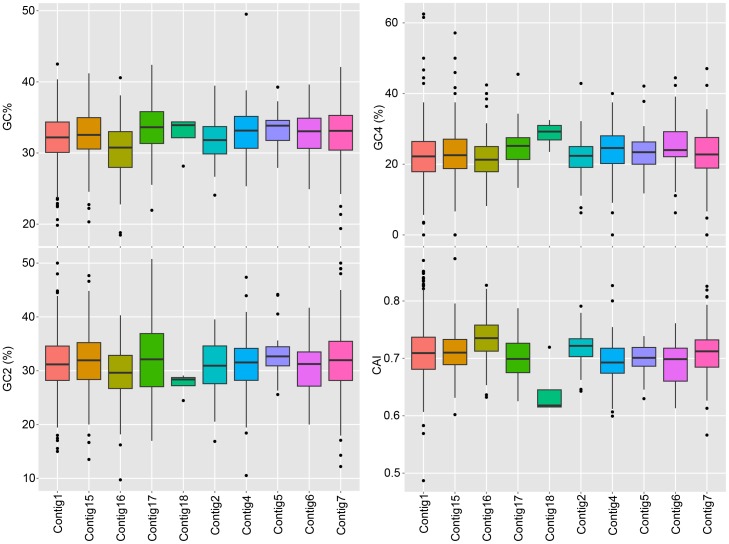
The ten contigs assigned to the *Ca.* X. pacificiensis genome from the metagenomic assembly have similar nucleotide composition. Boxplots are shown comparing GC% (top left), GC2 (bottom left), GC4 (top right) and CAI (codon adaptation index [49], bottom right) in contigs assembled from metagenomic sequence obtained from *L. patella* animal L6 and assigned to *Ca.* X. pacificiensis. The sizes of the contigs are shown in Table 6. Only Contig16 was found to have a statistically significant difference in nucleotide composition to any other contig (see Main Text, Table S2).

**Figure 13:**
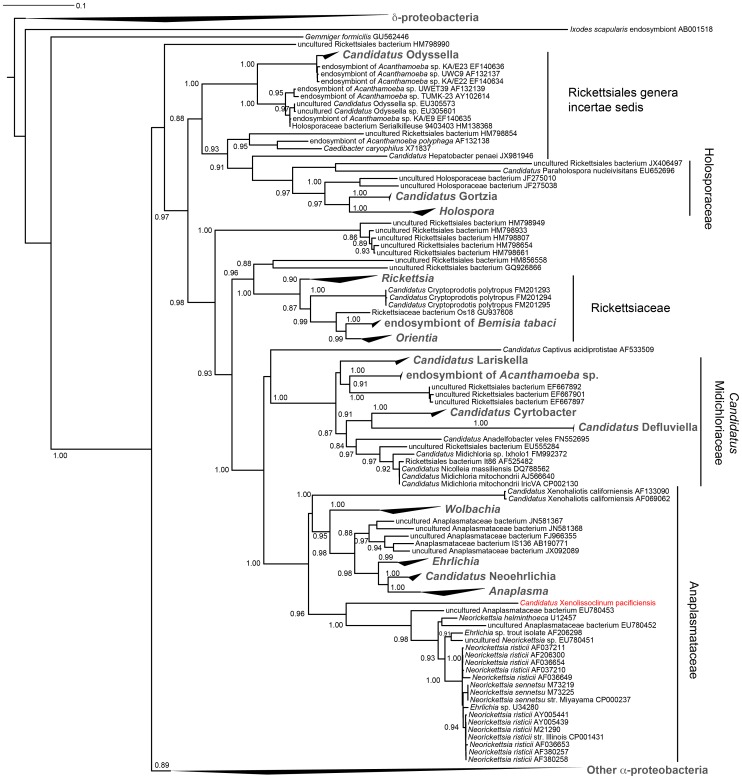
*Ca.* X. pacificiensis is a divergent member of the Anaplasmataceae family of the order Rickettsiales. The phylogenetic tree is based on 16S rRNA gene sequences, showing the Rickettsiales expanded, with the position of *Ca.* X. pacificiensis highlighted in red. Select *δ*-proteobacteria are included as an outgroup.

We previously found that the *L. patella* animal L6, despite being closely related to L5, did not contain *Ca.* E. faulkneri or the patellazoles by LCMS [13]. The remaining microbiome was very similar in L5 and L6, allowing us to determine that *Ca.* E. faulkneri was the likely source of the patellazoles. However, in 16S analysis, one group of sequences was present in L6, but *not* L5. These were assigned to the Anaplasmataceae family of *α*-proteobacteria. Shotgun metagenomic sequencing and assembly of DNA sequence derived from the zooids of L6 revealed ten high-coverage contigs (see Table 6) containing predicted genes that in BLASTP searches against NR aligned to homologs in Anaplasmataceae. We examined the nucleotide composition of the annotated coding regions in these contigs. The mean values and ranges for GC%, GC2, GC4 and codon adaptation index (CAI) [49] appeared to be similar for all contigs (see Fig. 12). We tested the statistical significance of the differences between contigs by ANOVA followed by Tukey’s honest significant difference (HSD) test. Only Contig16 was significantly different to any other contig with *p*<0.05 (see Table S2). All other pairwise tests were nonsignificant, indicating similar nucleotide composition in the remaining contigs. Importantly, the complete 16S, 5S and 23S rRNA genes were assembled within those contigs (see Table 6). In line with other Rickettsiales, the 16S gene is separated from the 5S and 23S rRNA genes. This separation is believed to have occurred some time after the split of ancestral Rickettsiales and mitochondrial lineages [50]. The 16S rRNA gene is highly divergent from known sequences, with the closest relative sharing only 84% identity (*Ehrlichia* sp. “trout isolate”, accession AF206298). Our own phylogenetic analysis shows that this bacterium is part of the Anaplasmataceae family, but diverges significantly from other genera (see Fig. 13), including another highly-divergent proposed species, *Ca.* Xenohaliotis californiensis [51]. Interestingly, the latter is a pathogen of abalone that causes withering syndrome [51], and is one of the few described marine species in this family of *α*-proteobacteria. Together with our findings, this suggests that there may be other highly divergent Anaplasmataceae lineages in marine environments. We term the new species *Candidatus* “Xenolissoclinum pacificiensis”, to reflect that it is likely not a long-term symbiont of *L. patella*.

**Table 6:** Contig lengths in the assembly of *Ca.* X. pacificiensis.

Contig	Length (bp)	Notes
Contig1	410,650	Contains 16S rRNA gene
Contig2	36,365	
Contig4	92,279	
Contig5	28,937	
Contig6	53,060	
Contig7	166,431	Contains 5S and 23S rRNA genes
Contig15	206,567	
Contig16a	174,382	
Contig17	38,820	
Contig18	6,336	

a Contig16 was found to have different nucleotide composition compared with other contigs with *p*<0.05, and thus was excluded from the final assembly (see Main Text).

**Figure 14:**
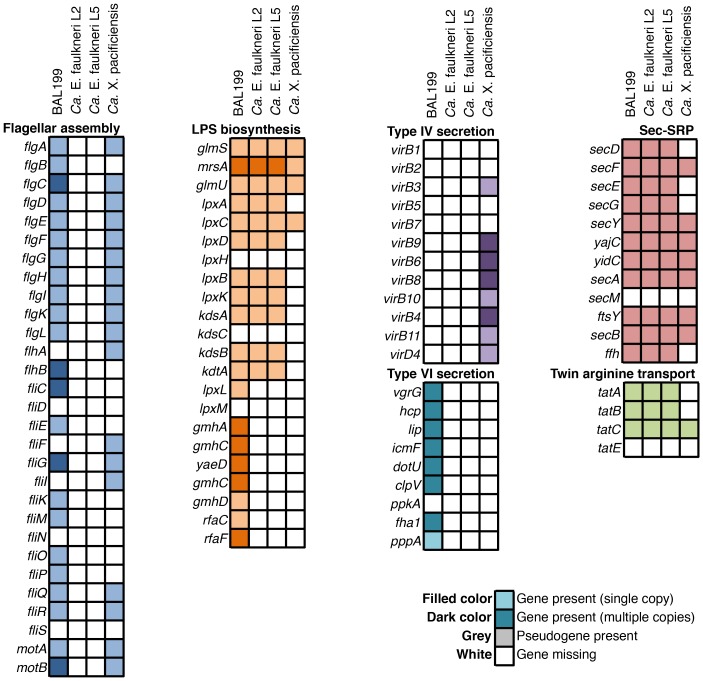
*Ca.* X. pacificiensis possesses genes involved in various pathogenicity-related processes, while *Ca.* E. faulkneri lacks genes in these categories.

The genome of *Ca.* X. pacificiensis is smaller than those of *Ca.* E. faulkneri, but it has greater coding density (77.2% vs. 56–57%, see Table 1). Examination of the genes present in the genome of *Ca.* X. pacificiensis indicate that it is in a less advanced state of genome reduction, compared with *Ca.* E. faulkneri. *Ca.* X. pacificiensis possesses *recA*, maintains the majority of additional genes required for homologous recombination, and the genome still contains prophage and transposon sequences (see Fig. 5). *Ca.* X. pacificiensis also contains a homolog of the *comEC* gene, and may be competent for DNA uptake [52]. Unlike *Ca.* E. faulkneri, *Ca.* X. pacificiensis retains key genes involved in chromosome replication and cell division - *dnaA*, *ftsK* and *ftsZ*, among others. For example, it possesses *parA* and *parB*, which are involved in chromosomal segregation during division [53] and are not found in *Ca.* E. faulkneri. *Ca.* X. pacificiensis also possesses the genes *mutS* and *mutL*, suggesting that it is able to carry out mismatch repair. Like other Anaplasmataceae, *Ca.* X. pacificiensis lacks a functional LPS biosynthetic pathway [37] (see Fig. 14), but unlike the majority of this family, it maintains many genes involved in flagellar assembly [54].

**Figure 15:**
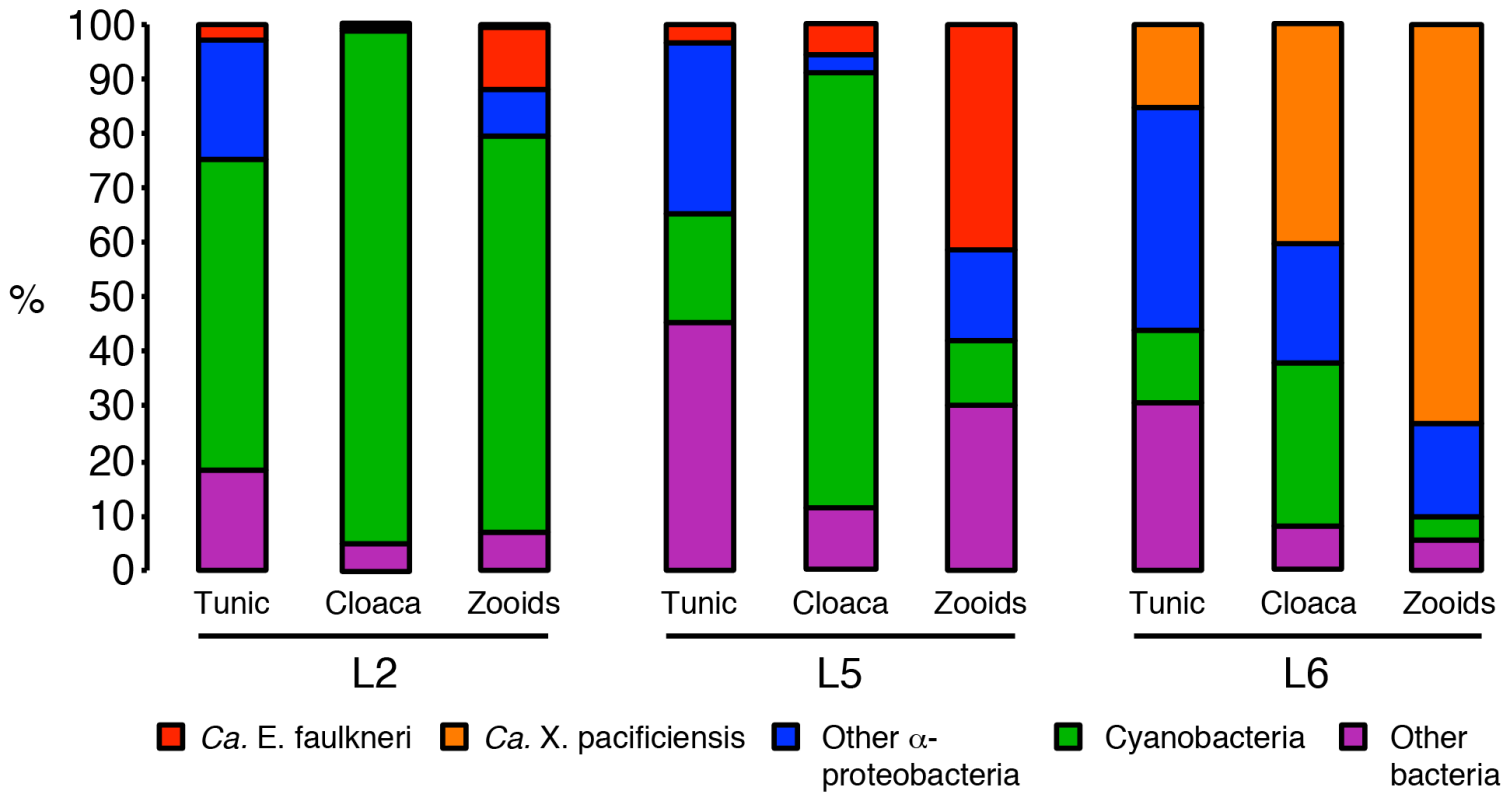
Microbiome variation in different tissues of *L. patella* animals L2, L5 and L6, determined by 454 16S rRNA gene amplicon sequencing. The content of *Ca.* E. faulkneri (red) and *Ca.* X. pacificiensis (orange) 16S rRNA is shown (in each case defined as sequences ≥98% identity to the 16S sequence in the relevant assembled genome).

Many bacteria in the family Anaplasmataceae are obligate intracellular pathogens that infect mammals through arthropod vectors, such as ticks [37]. For instance, *Neorickettsia risticii* causes Potomac horse fever [55], and *Ehrlichia chaffeensis* was recently found to cause disease in humans [37]. *E. chaffeensis* is found in vacuoles in white blood cells (monocytes, macrophages and dendritic cells), where it actively divides and evades phagocytic destruction [37]. Likewise, mammalian infection with other Anaplasmataceae, such as *N. risticii* can manifest through intracellular infiltration of white blood cells [37]. We previously visualized *Ca.* E. faulkneri cells within tunicate blood cells with the amorphous and granular appearance of phagocytes [13], suggesting that in *L. patella* animal L6, *Ca.* X. pacificiensis could have displaced *Ca.* E. faulkneri by colonizing the same cell type. In analysis of 16S amplicon data from different tissues of L6, we found an enrichment of the *Ca.* X. pacificiensis 16S rRNA gene sequence in the zooids versus the tunic or cloacal contents (see Fig. 15), suggesting that *Ca.* X. pacificiensis may have similar tissue localization to *Ca.* E. faulkneri. Further studies would be required in order to determine whether *Ca.* X. pacificiensis resides within the same cell type as *Ca.* E. faulkneri. Interestingly, we found a very small number of sequences within 98% identity of the *Ca.* X. pacificiensis 16S rRNA sequence in animal L2 (2 and 25 sequences in the cloacal contents and zooids out of 7,775 and 5,477 sequences, respectively). This suggests that low-level infection of *Ca.* X. pacificiensis may be common in this clade of *L. patella*, and that overt infection only occurs in susceptible individuals.

### Genome Analysis Reveals *Ca.* Endolissoclinum faulkneri and *Ca.* X. pacificiensis have Different Lifestyles

Both long term vertically-transmitted symbionts and intracellular pathogens go through a process of genome reduction [1], [56]. For instance, both *Ca.* E. faulkneri and *Ca.* X. pacificiensis are deficient in amino acid biosynthesis pathways, due to availability of amino acids in the intracellular environment. However, while *Ca.* E. faulkneri possesses the *ptz* pathway for the patellazoles, which likely serve as chemical defenses for the host animal, we did not find a compelling symbiotic function for *Ca.* X. pacificiensis. Like many other members of the family Anaplasmataceae, *Ca.* X. pacificiensis is either a pathogen of *L. patella*, or the tunicate acts as a reservoir for infection of other hosts, passed through an unknown vector.

Unlike *Ca.* E. faulkneri, *Ca.* X. pacificiensis is likely able to control its own replication and division, and is competent in DNA repair and recombination. In the absence of an advantageous function for the host, *Ca.* X. pacificiensis is at best a parasite, but it also contains several features that suggest it adopts a pathogenic lifestyle. For instance, although most *Ehrlichia* do not possess flagellar assembly genes, they have been shown to play a role in the growth of another intracellular pathogen, *Legionella pneumophila* [57]. Additionally, *Ca.* X. pacificiensis possesses a type IV secretion apparatus, which has been implicated in the pathogenesis of *Ehrlichia* and other Anaplasmataceae infections [56]. The genes in this apparatus (see Fig. 14) are in line with other members of the Anaplasmataceae, which commonly lack homologs of *virB1*, *virB2*, *virB5* and *virB7* [56]. In *E. chaffeensis* infection, tandem repeat- and ankyrin repeat-containing proteins are thought to play a major role in host interactions during infection [37]. Ankyrin repeats are rare in prokaryotes [50], and in the context of *Ehrlichia* infection these domains are thought to influence host gene expression [37]. We found one ankyrin repeat protein (P857_417) in the genome of *Ca.* X. pacificiensis, which was predicted to be secreted through a type IV pathway with SSPred [58], suggesting that this protein is a secreted effector in infection.

In some insect systems, the host is thought to tightly control the proliferation of intracellular symbionts [5]. Limited division is one of the hallmarks of symbiosis in contrast to intracellular infection [5]. Therefore the loss of many central replication and division genes in *Ca.* E. faulkneri distinguishes it as a symbiont in comparison to *Ca.* X. pacificiensis, in addition to the presence of the *ptz* pathway for the protective patellazoles. From our gene inventory analysis we conclude that *Ca.* X. pacificiensis is able to control its own replication and division. In addition, it shares several features common to intracellular pathogens in the family Anaplasmataceae. The known cellular habitats of pathogens such as *E. chaffeensis* and our own 16S amplicon data suggest that *Ca.* X. pacificiensis may have displaced *Ca.* E. faulkneri by taking residence in the same host tissue. Similar phenomena have been observed in insects, where occasional loss of long-term symbionts is observed in specific insect lineages [14]. Presumably this displacement comes at a cost for animal L6. If *Ca.* X. pacificiensis commonly infects and displaces symbionts in this *L. patella* population, it may eventually adapt to a symbiotic lifestyle, similar to some *Wolbachia* species [59], [60]. *Ca.* X. pacificiensis may be capable of accepting horizontally transferred DNA and thus might be able to take on functions that replace the *ptz* pathway of *Ca.* E. faulkneri.

## Conclusion

While the progress of genome decay in intracellular symbionts is most well studied in insects, our results show that such phenomena are not limited to this group of hosts. Insect symbionts most often provide nutritional support by synthesizing essential nutrients not found in the insect’s diet, and are sometimes found to be protective against parasites or infection [1]–[3]. The symbiotic relationship of *Ca.* E. faulkneri and *L. patella* is instead based on the production of relatively large natural products, and our analysis suggests that these products have been important enough to be conserved against a drive of genome reduction for millions of years. Evidence of such a stable symbiotic relationship cements the role of symbiotic bacteria in natural product production [61], [62], and suggests that intracellular symbionts may be an important source of natural products.

## Materials and Methods

### Collection, DNA Extraction, Isolation and Sequencing

Permission to perform field research was granted by the Papua New Guinea Department of Environment and Conservation and facilitated by the University of Papua New Guinea. Animals were collected as described previously [13] by scuba. Zooids were dissected from L5 and L6 as described previously [13] from samples that had been preserved in RNAlater. DNA was then extracted using a standard method for tunicates [63], followed by repurification using the Genomic DNA Clean & Concentrator kit (Zymo Research). Double-stranded DNA was quantified using the Quant-it PicoGreen kit (Invitrogen), before being subjected to sequencing on an Illumina HiSeq 2000 in a 101 bp paired-end run (one complete lane for each sample). Before assembly, reads were filtered to remove read pairs that did not have quality scores ≥30 over more than 40 bp on each direction.

### COXI Identity Matrix (Fig. 2)

The COXI tree was described previously [13]. Briefly, nucleotide sequences were aligned with ClustalW-MPI [64]. The alignment was inspected manually using ClustalX [65], and any sequences that were particularly short or unilaterally introduced large inserts into the alignment were discarded. The alignment was trimmed using a Perl script (trim_aligned_fasta.pl [13]), and then used as an input for FastTree [66], using the parameters -slow -spr 5 -mlacc 3 -gamma -gtr -nt. Identity values presented here were calculated by re-examining the alignments used to make the tree using a Perl script (identity_matrix.pl [13]). To make the figures, values were imported into Microsoft Excel, where a continuous color scale running from dark green (lowest value in figure) to bright red (highest value in figure) was applied.

### Assembly of the *Ca.* E. faulkneri L5 Genome

An initial assembly was constructed using Velvet [67] with a k-mer size of 61 bp and a coverage cutoff of 5. Protein sequences from the genome of *Ca.* E. faulkneri L2 were used as queries for a TBLASTN search against the raw assembly. Hit contigs with a % identity to the queries of ≥60% were extracted and aligned to the *Ca.* E. faulkneri L2 genome. Short contigs that did not align to the L2 genome were rejected, leaving a set of 63 contigs with a combined size of 1.51 Mbp and an n50 of 37.4 kbp. These contigs were used as queries in a BLASTN search against the raw Illumina reads, using an e-value cutoff of 1×10^−30^. Hit reads were extracted and assembled with Velvet [67] using a k-mer size of 67 bp and an expected coverage of 25 to give an assembly of 5 contigs with total size 1.52 Mbp. These were used as queries in another round of BLASTN searching against the raw reads and subsequent assembly. After removal of runs of Ns with a custom Perl script (fasta_split_Ns.pl, Text S1), contigs from the two iterative assemblies were assembled in Sequencher to give an assembly of 4 contigs totaling 1.51 Mbp. The remaining regions were amplified by PCR (see Table 7) using Platinum Taq High Fidelity (Invitrogen), cloned using the TOPO TA cloning kit (Invitrogen) and sequenced to yield the complete chromosome of *Ca.* E. faulkneri L5. In order to validate the assembly, the entire set of filtered Illumina reads were aligned to the assembled sequence with Bowtie 2 [68]. Unaligned reads were removed from the resulting sam file using a Perl script (sam_remove_unaligned.pl, Text S2). The resulting sam file was converted to a bam file using Samtools [69], before variants were called (also using Samtools). 96 variant sites were found, indicating a low error rate (0.0063%). For comparison, the same procedure was carried out with reads used to assemble *Ca.* E. faulkneri L2. This found a similar amount of variants - 34 (0.0023%). The vcf file generated in this analysis are available in the supporting information (see Text S3).

**Table 7:** Primers used in this study.

Name	Sequence	Reference	Notes	5′ coordinatea
ef33_734_Rout_2	CGTAGACCATAACGCAGTATGG	This study	Contig joining	702,249
ef33_738_Lout_2	CGCAAGCACTAAACGGCAC	This study	Contig joining	705,184
ef33_1327_Rout	CGTCTATTAAAGGCTGCATCGG	This study	Contig joining	1,262,269
ef33_1329_Lout	GGTTCGCATGAGCTTGTATCG	This study	Contig joining	1,265,356
ef33_829_Rout	TCCACCACTGACATTAGTAGATAC	This study	Contig joining	798,954
ef33_832_Lout	CAGTACAGCGCGAGATACAATG	This study	Contig joining	802,162
ef33_124_Lout	ACACCTTCAGAACTTACAGCAATG	This study	Contig joining	123,860
Thal16SFrev	AGGGTGTGGTTATTGGAGAC	This study	Contig joining	117,880
Thal_16S-F	GTCTCCAATAACCACACCCT	Kwan *et al.*	Contig joining	117,899
		2012 [13]		
Thal_16S-R	GCTTTCGATACTGCATAGCTC	Kwan *et al.*	Contig joining	117,512
		2012 [13]		
Thal_23S_R	CCTCACGGTACTAGTTCAC	Kwan *et al.*	Contig joining	119,618
		2012 [13]		
Supercontig_9-	GAATAATTGATACTCCAGCTAGC	Kwan *et al.*	Contig joinint	114,009
R-out		2012 [13]		
27F	AGAGTTTGATCCTGGCTCAG	Weisburg *et al.*	16S	116,943
		1991 [80]	amplification	
1492R	GGTTACCTTGTTACGACTT	Reysenbach *et al.*	16S	118,390
		1992 [81]	amplification	
M13_4_TOPO-F	GTAAAACGACGGCCAG	Supplied with	TOPO clone	
		TOPO-TA kit	sequencing	
M13_4_TOPO-R	CAGGAAACAGCTATGAC	Supplied with	TOPO clone	
		TOPO-TA kit	sequencing	
ef33_829_insert_F	TTGTTAAGTGCATCATGCACCG	This study	Sequencing	800,604
ef33_832_insert_R	AACAGCAAAACTCAGATAATG	This study	Sequencing	801,342
ef33_1327_insert_F	ATGAGATAACTGGATACACGC	This study	Sequencing	1,263,989
ef33_1329_insert_R	TTAGTGTAGGAGTAGTATTGTTC	This study	Sequencing	1,264,424
ef33_734_insert_F	TCAATCAACAAGAGAGGAGTAC	This study	Sequencing	703,427
ef33_738_insert_R	AAGACGTCATTTGGCATAGTTAGG	This study	Sequencing	704,265
ef33_23Sreg_124F2	ATTCAAATAGCAACCGGGATAAC	This study	Sequencing	123,190
ef33_23Sreg_124F3	TAAGGATAATCTGCCATACATAAC	This study	Sequencing	122,527
ef33_23Sreg_124R1	CCTGACTGCGAGGCTGAC	This study	Sequencing	121,371
ef33_23Sreg_C2F2	CTACCCCCTACTCTGTGCC	This study	Sequencing	120,807
ef33_23Sreg_R1	GAGGTCAGCTTACAAGAAAACCG	This study	Sequencing	118,477

a The coordinates shown refer to the chromosome of *Ca.* E. faulkneri L5.

### Assembly of the Draft *Ca.* X. pacificiensis Genome

An initial assembly was carried out using Meta-Velvet [70] with a k-mer size of 61 bp, and examined manually. Several contigs with k-mer coverage ∼30× seemed to contain genes related to Anaplasmataceae family *α*-proteobacteria. To isolate the genome of this organism, an assembly was carried out using Velvet [67] with the parameters -cov_cutoff 22 -exp_cov 30 -max_coverage 40. Contigs larger than 10 kbp were used as queries in BLASTN searches against the reads similarly to the assembly of *Ca.* E. faulkneri. Contigs from both the first and second round assemblies were manually filtered to remove tunicate contigs and assembled in Sequencher. The resulting assembly had 17 contigs and an n50 of 174.4 kbp. An initial annotation was constructed using Clovr [71] and loaded into a MySQL database using Ergatis [72]. Proteins from this annotation were used as queries in a BLASTP search against the NR database. The outputs from these searches were used to assign phylogeny with Megan [73]. Contigs were rejected if they did not contain a single gene assigned to the family Anaplasmataceae. The GC content, GC2, GC4 and CAI for all predicted protein-coding ORFs were calculated using Perl scripts (genbank_multi_process.pl [Text S4], CAI_calculate.pl [13]). For CAI calculations, genes from Contig1 (which contains the complete 16S rRNA gene) were used to calculate reference RCSU and *w* values [67]. The results of these calculations were plotted as boxplots in R, using the ggplot2 package. ANOVA analysis was carried out in R, using the aov function, followed by the TukeyHSD test for significance (see Table S2).

### Annotation of Bacterial Genomes

The contigs of *Ca.* X. pacificiensis and the complete *Ca.* E. faulkneri L5 chromosome were annotated with Clovr [71], and then loaded into a MySQL database (Chado schema [74]) using Ergatis [72]. The databases were manually manipulated using the Manatee interface (http://manatee.sourceforge.net/igs/index.html).

### Identification of Homologs in L2, L5 and BAL199

L5 proteins were used as queries in a BLASTP search against L2 proteins with default parameters and an e-value cutoff of 10 to identify homologs. The hits were processed according to the following algorithm.

1. Retain only best (lowest e-value) hit (subject) for each query.
2. Order hits in ascending order of e-value.
3. Discard pairs where subject occurs a second and subsequent times.
4. Discard pairs where query and subject produce dissimilar hit lists in BLASTP searches against NR. (First 10% in descending e-value list checked).
5. With the assumption that L2 and L5 are syntenic, calculate the expected coordinate in L5 for every gene in L2 that still exists in the list. For each gene calculate the difference between expected and observed coordinate in L5.
6. Order the hits in descending order of absolute difference between the expected and observed coordinate in L5. Examine each pair manually in the manner of point 4 (First 10% in list examined).
7. Order the hits in descending order of absolute length difference between genes in L2 and L5. Move start site back in the shorter gene if homology extends to an earlier start codon (as determined by TBLASTN searches using the longer gene as a query against the whole genome of the strain containing the shorter gene).
8. Orphan genes are genes in L2 and L5 that do not have a paired gene in the list resulting from this process.

Orphan genes were examined with care, and hypothetical orphans with length less than 300 bp and no homology to any bacterial gene in BLASTP searches against NR were removed from the list of annotated genes. Both sets of homologs were then used as queries in BLASTP searches against BAL199 proteins in our previous annotation [13]. The results were examined manually to detect BAL199 annotation errors, and lower similarity hits were checked by searching the query against the NR database in a BLASTP search (default parameters). Homologs to BAL199 were deemed real when BAL199 was ranked in the top 100 hits against NR. The resulting hits were then examined using a Perl script that checked whether L2–L5 homologs aligned to the same BAL199 protein (BAL199_check.pl, Text S5). The coordinates of homolog pairs in L2 and L5 were used to plot Figure 4B using the ggplot2 package in R. The lengths of the corresponding homolog pairs were also used to plot Figure 10 using the ggplot2 package in R.

### Intergenic Synteny Analysis in *Ca.* E. faulkneri

Intergenic sequences in *Ca.* E. faulkneri strains L2 and L5 were extracted from GenBank files obtained directly from Manatee using a Perl script (genbank_multi_process.pl, Text S4). Intergenic sequences smaller than 100 bp were removed. The resulting sequences from L2 were used as queries in BLASTN and TBLASTX searches against a blast database of the intergenic sequences in L5 using default parameters, i.e. an e-value cutoff of 10. The top hit for each query in the BLASTN search was used to determine the coordinates of the points in Figure 4C, which was plotted using the ggplot2 package in R. Because TBLASTX searches typically yield many duplicate hits between the same query and subject corresponding to different reading frames, a Perl script was used (check_blastx_results.pl, Text S6) to group together these hits and calculate the average % identity weighted by length of alignment between intergenic sequence pairs. The midpoint coordinate for each intergenic sequence in its corresponding genome was used to plot figure 4D.

### Identification of Pseudogenes in *Ca.* E. faulkneri Strains

Pseudogenes were detected in each *Ca.* E. faulkneri strain using TBLASTN with default settings by querying the orphan genes from one strain against a database of the intergenic sequences not assigned to genes in the corresponding strain. Hits that were classified as pseudogenes had the following characteristics:

1. Several hits for the same query localized to an area not larger than the query sequence in the subject genome.
2. The hits are in a locus within the subject genome consistent with the conservation of synteny observed in other analyses.

In order to detect pseudogenes that were potentially degraded in both strains, remaining intergenic regions larger than 100 bp were used as BLASTX queries against the entire NR database (default settings). Hits with e-values lower than 1×10^−3^ against one or more bacterial nonhypothetical genes were considered pseudogenes. Intergenic sequences that contained identified pseudogenes were removed from the intergenic set of sequences, then the GC percent of sequences in the sets of pseudogenes, intergenic sequences, coding sequences and RNA genes in both strains of *Ca.* E. faulkneri were tabulated. ANOVA analysis and the TukeyHSD test were carried out in R, as with the analysis of *Ca.* X. pacificiensis contigs (vide supra). It was found that the intergenic, coding sequences and RNA genes in the respective strains were not significantly different, so these groups were combined. The pseudogenes from each strain were also combined into a single group, since the small sample size of each strain individually would limit the detection of statistical differences. ANOVA and the TukeyHSD test were then repeated in R, to give the *p* values reported in Figure 8.

### Analysis of Gene Inventories

The presence and absence of genes for figures 5 and 14 was assessed a number of ways. Protein sequences from *Ca.* E. faulkneri L2 and L5, BAL199 and *Ca.* X. pacificiensis were used as queries in a BLASTP search against the NR database (default parameters), with -max_target_seqs set to 200. The resulting blast tables were imported into Megan [73], and their functional annotations were viewed in the KEGG analyzer section of the program. The automatic annotations generated by Clovr [71] were also used, as were manual annotations made in Manatee.

### Divergence Comparisons (Figure 6)

Protein coding genes of the bacterial genomes were extracted from GenBank files using a Perl script (genbank_multi_process.pl, Text S4). Homologous genes in the strain pairs were determined as with *Ca.* E. faulkneri L2/L5/BAL199, described above. The % identities for each homolog pair were tabulated and imported into R, where the distribution of identities was plotted as a density plot with the ggplot2 package. Plots were exported as SVG files and manually normalized so that the maximum value was the same perpendicular distance from the *x*-axis in Adobe Illustrator. The accession numbers of the genomes used were: *Buchnera aphidicola* APS: NC_002528, *Buchnera aphidicola* Sg: NC_004061, *Sulcia muelleri* GWSS: NC_010118, *Sulcia muelleri* SMDSEM: NC_013123, *Brucella melitensis* bv. 1 str. 16M: NC_003317, *Brucella ovis* ATCC 25840: NC_009504.

### Construction of Genome Figures 3 and 7

Genome figures were constructed using Circos [75], using scripts described previously [13]. For Figure 7, the coordinates of stop codons in three frames were generated with a Perl script (Make_start_stop_heatmap_tracks.pl, Text S7, which uses an additional Perl module: Text S9), and the inner L2 genome representation was added later in Adobe Illustrator.

### Comparison of Divergence Amongst Related Natural Product Biosynthetic Pathways

Biosynthetic gene sequences used for Figure 11 were retrieved from NCBI and the relevent groups were aligned with Clustal-Omega [76]. Protein alignments were constructed first, and then nucleotide alignments were constructed from these using a Perl script (nucleotide_translation_alignment.pl, Text S8). Identity values and shading were calculated as with the Figure 2. The accession numbers of the pathway records used were: ET-743 pathway: HQ609499, safracin pathway: AY061859, saframycin A pathway: DQ838002, saframycin Mx1 pathway: MXU24657, pederin pathway: AY328023, AY426537, onnamide pathway: AY688304, bryostatin “Deep”: DQ889941, DQ889942, bryostatin “Shallow”: EF032014.

### 16S rRNA Gene Amplicon Sequencing by 454 and Analysis

Amplification of 16S rRNA gene sequences and sequencing by 454 pyrosequencing was carried out as previously described [13]. Broad categorization was carried out using the Clovr-16S pipeline [71]. The *Ca.* E. faulkneri L2 and L5 16S rRNA gene sequences obtained from assembly and Sanger sequencing were used as queries in a BLASTN search (default parameters) against databases containing the raw 454 reads. Reads were classified as belonging to *Ca.* E. faulkneri or *Ca.* X. pacificiensis when they shared ≥98% identity with the relevant query sequence. Both *Ca.* E. faulkneri sequences were used as queries against L6 samples, but no reads above the identity threshold were found.

### Analysis of Genome Size, Number of Genes and Presence of *ftsZ* and *dnaA* among Intracellular Bacteria (Figure 9)

Complete genomes for intracellular bacteria were downloaded from NCBI as GenBank files and processed with a Perl script (genbank_multi_process.pl, Text S4). The genome sizes and number of protein coding genes were taken directly from tabulated outputs of the script, and the presence or absence of annotated *ftsZ* and *dnaA* genes was confirmed manually. The accession numbers of the genomes used are included in the Supporting Information (Table S1).

### Construction of the *α*-proteobacterial Phylogenetic Tree (Figure 13)

The 16S rRNA gene sequence for *Ca.* X. pacificiensis was uploaded to the Ribosomal Database Project webserver [77], and additional sequences were selected from the RDP database. Good quality sequences that were ≥1200 bp from type strains classified as *δ*-proteobacteria were selected to act as an outgroup. Good quality type strain sequences ≥1200 bp were also selected from the following *α*-proteobacterial orders: Rhodobacterales, Caulobacterales, Kordiimonadales, Kiloniellales, Parvularculales, Rhizobiales, Sphingomonadales, Rhodospirillales and Sneathiellales. Additionally both type and nontype strain sequences were selected from Rickettsiales. Collectively, these sequences were downloaded as an aligned fasta from RDP, and the alignment was manually inspected in ClustalX. Sequences that appeared to unilaterally cause large insertions in the alignment were discarded, and the alignment was then trimmed using a Perl script (trim_aligned_fasta.pl [13]). The phylogenetic tree was constructed using FastTreeMP [66], using the parameters -slow -spr 5 -mlacc 3 -gamma -gtr -nt. The tree was rooted and manipulated using the Interactive Tree of Life webserver [78].

## Accession Numbers

The genome sequence data for *Ca.* E. faulkneri L5 and *Ca.* X. pacificiensis L6 have been submitted to the National Center for Biotechnology Information (NCBI) (http://www.ncbi.nlm.nih.gov). The accession numbers are CP006745 for *Ca.* E. faulkneri L5 and AXCJ00000000 for *Ca.* X. pacificiensis.

## Supporting Information

Table S1 **Accession numbers of genomes used to construct** **Figure 9** **. (12K XLSX).**
(XLSX)

Table S2 **Calculated** ***p*** **values in pairwise comparisons of nucleotide composition parameters (GC%, GC2, GC4, CAI) in putative contigs of** ***Ca.*** **X. pacificiensis, resulting from ANOVA followed by TukeyHSD analysis. (67K XLSX).**
(XLSX)

Text S1 **Perl source code for fasta_split_Ns.pl. (1.2K PL).**
(PL) Click here for additional data file.

Text S2 **Perl source code for sam_remove_unaligned.pl. (1.0K PL).**
(PL) Click here for additional data file.

Text S3 **VCF file of the variants called by Samtools from the Illumina read alignment to the** ***Ca.*** **E. faulkneri L5 assembly. (19K VCF).**
(VCF)

Text S4 **Perl source code for genbank_multi_process.pl. (46K PL).**
(PL) Click here for additional data file.

Text S5 **Perl source code for BAL199_check.pl. (2.4K PL).**
(PL) Click here for additional data file.

Text S6 **Perl source code for check_blastx_results.pl. (2.1K PL).**
(PL) Click here for additional data file.

Text S7 **Perl source code for Make_start_stop_heatmap_tracks.pl. (4.7K PL).**
(PL) Click here for additional data file.

Text S8 **Perl source code for nucleotide_translation_alignment.pl. (2.9K PL).**
(PL) Click here for additional data file.

Text S9 **Perl module Sequence_toolkit.pm, used in Make_start_stop_heatmap_tracks.pl. (47K PL).**
(PM) Click here for additional data file.
